# Analysis of risk factors for neurological symptoms in patients with purely hepatic Wilson’s disease at diagnosis

**DOI:** 10.1186/s12883-023-03105-w

**Published:** 2023-02-28

**Authors:** Sheng-Peng Diao, Yang-Sha Zhuang, Ye-Qing Huang, Zhi-Hua Zhou, Ai-Qun Liu, Ming-Fan Hong

**Affiliations:** 1 Department of Neurology, The First Affiliated Hospital, Jinan University, Guangzhou, 610630 Guangdong China; 2 Department of Neurology, College of Clinical Medicine, The First Affiliated Hospital, Guangdong Pharmaceutical University, Guangzhou, 510062 Guangdong China

**Keywords:** Wilson’s disease, Neurological symptoms, Hepatic, Penicillamine, Zinc gluconate

## Abstract

**Objective:**

To analyze and explore the risk factors for neurological symptoms in patients with purely hepatic Wilson's disease (WD) at diagnosis.

**Methods:**

This retrospective study was conducted at the First Affiliated Hospital of the Guangdong Pharmaceutical University on 68 patients with purely hepatic WD aged 20.6 ± 7.2 years. The physical examinations, laboratory tests, color Doppler ultrasound of the liver and spleen, and magnetic resonance imaging (MRI) of the brain were performed.

**Results:**

The elevated alanine transaminase (ALT) and aspartate transaminase (AST) levels and 24-h urinary copper level were higher in the purely hepatic WD who developed neurological symptoms (NH-WD) group than those in the purely hepatic WD (H-WD) group. Adherence to low-copper diet, and daily oral doses of penicillamine (PCA) and zinc gluconate (ZG) were lower in the NH-WD group than those in the H-WD group. Logistic regression analysis showed that insufficient doses of PCA and ZG were associated with the development of neurological symptoms in patients with purely hepatic WD at diagnosis.

**Conclusion:**

The development of neurological symptoms in patients with purely hepatic WD was closely associated with insufficient doses of PCA and ZG, and the inferior efficacy of copper-chelating agents. During the course of anti-copper treatment, the patient's medical status and the efficacy of copper excretion should be closely monitored.

## Introduction

Hepatolenticular degeneration (HLD), also known as Wilson's disease (WD), is an autosomal recessive disorder of copper metabolism. The prevalence of this disease is 1/26000 ~ 1/30000 globally, and the population carrier rate is approximately 1/90 [1, 2]. The abnormal WD gene is localized in the long arm of chromosome 13q14.3. This gene encodes P-type adenosine triphosphatase (ATP7B) that is copper transporter [3]. Mutated ATP7B causes the functional loss of a copper transporter, resulting in the decreased synthesis of ceruloplasmin and impairment of hepatic biliary copper excretion. This leads to the progressive copper deposition in the liver and other organs, such as brain, kidneys, and cornea. Clinical manifestations of this disease include hepatic, neurological, and psychiatric signs and symptoms [4].

Symptomatic patients with WD are classified as hepatic, neurological, or mixed depending on their clinical presentation, liver imaging, liver enzymes, and magnetic resonance imaging (MRI) of the brain [5]. The majority of patients with WD present with hepatic and neurological symptoms. Liver involvement is more common in WD, with either clinically asymptomatic or symptomatic liver involvement, and when WD patients with hepatic symptoms are not diagnosed and treated timely, they may develop neurological symptoms, such as tremor, slurred speech, and drooling [5, 6]. Neurological symptoms are tricky in the therapy of WD, and the therapeutic efficacy is mainly poor. How to more effectively prevent the development of neurological symptoms and identify the high-risk factors for neurological symptoms has noticeably attracted clinicians' attention. The present study aimed to analyze 14 patients with purely hepatic WD symptoms who developed neurological symptoms during follow-up and to evaluate the risk factors for neurological symptoms.

## Study subjects and methods

### Study subjects

A total of 68 patients with purely hepatic WD without neurological symptoms who were diagnosed and treated in the Department of Neurology of the First Affiliated Hospital of Guangdong Pharmaceutical University (Guangzhou, China) from January 2016 to December 2021, were recruited from 65 families, including 35 men and 33 women who aged 20.6 ± 7.2 years old. All patients were followed up for more than one year. During the follow-up, neurological symptoms were assessed in 14 patients.

Patients were divided into two groups, including the H-WD group (i.e., the presence of hepatic abnormalities and absence of neurological symptoms) and hepatic WD with neurological symptoms at follow-up (NH-WD) group. The present study was approved by the Ethics Committee of the First Affiliated Hospital of Guangdong Pharmaceutical University. All patients signed the informed consent forms.

### Diagnostic criteria and exclusion criteria

All patients met the diagnostic criteria for WD [7], including ceruloplasmin < 0.2 g/L, 24-h urinary copper > 100 μg, and corneal Kayser-Fleischer (K-F) ring positivity. The exclusion criteria were as follows: 1) complication with liver failure; 2) patients with neurological symptoms in the past and symptoms disappeared before the first follow-up; 3) stress response to traumatic, surgical, or emotional events; and 4) patients who did not refer to the hospital regularly for recheck and had incomplete data.

### Physical and laboratory examinations

All patients' medical records were collected, and they underwent related physical examinations and routine blood tests, in which the levels of blood alanine transaminase (ALT), blood aspartate transaminase (AST), serum copper, serum ceruloplasmin, and 24-h urinary copper were measured. All patients underwent a color Doppler ultrasound of the liver and spleen and an MRI of the brain during follow-up.

Serum copper and 24-h urinary copper levels were determined by the flame method using a Hitachi Z-5000 nuclear absorption spectrophotometer (Hitachi, Tokyo, Japan).

All WD patients underwent 3.0 T GE Silent MR scanning, axial T1-weighted imaging (T1WI) [repetition time (TR)/echo time (TE) = 3600 ms/100 ms], and T2-weighted imaging (T2WI) [TR/TE] = 3600 ms/100 ms], in which the layer thickness was 5.0 mm, and the layer spacing was 0.5 mm. Images were analyzed by two radiologists who had more than 10 years of experience in MRI of the brain.

### Treatment and follow-up

All patients were followed up for at least 1 year and were evaluated by routine examinations every year. The mean follow-up time was 48.9 (range, 12–72) months. Penicillamine (PCA) and zinc gluconate (ZG) were administrated as initial and maintenance therapies, respectively for all patients. PCA was administered 1–2 h before meals between 750 and 1250 mg daily, divided into 2–4 doses, and ZG was administered after meals between 210 and 420 mg daily, divided into 3 doses. The clinical, laboratory, and imaging data of all the patients were recorded and evaluated during follow-up.

### Statistical analysis

Statistical analysis was performed using SPSS 19.0 software (IBM, Armonk, NY, USA). Data were tested for homogeneity of variance, and normally distributed data were expressed as the mean ± standard deviation. The Howell method was used when the variance was unevenly distributed. Enumeration data were compared using the Chi-square test or Fisher's exact test, and the measurement data were compared using the independent t-test or Mann–Whitney U test. Logistic regression analysis was performed to identify risk factors for neurological symptoms. Statistical significance was set at *P* < 0.05.

## Results

### Baseline data

There was no significant difference between the H-WD and the NH-WD groups in terms of gender, age, course of disease, or history of jaundice (Table 1). Besides, 14 patients with purely hepatic WD developed neurological symptoms during follow-up, including 1, 1, 2, 3, 2, and 5 patients with follow-up periods of (0–6 months), (6–12 months), (1–2 years), (2–3 years), (3–4 years), (over 5 years), respectively after diagnosis.

**Table 1:** Comparison of clinical and laboratory data between the H-WD group and NH-WD group

Project	NH-WD(*n* = 14)	H-WD(*n* = 54)	*P*
Age (year)	19.9 ± 4.7	20.7 ± 7.8	0.63
Gender (male/female)	7/7	28/26	0.902
Course of disease (month)	52.5 ± 15.2	48.2 ± 14.8	0.347
History of jaundice (cases)	3	8	0.848
White blood cell count(× 10^9^/L)	4.97 ± 2.81	4.82 ± 2.62	0.853
Platelet count(× 10^9^/L)	120.23 ± 50.45	110.24 ± 72.07	0.63
Red blood cell count(× 10^12^/L)	4.24 ± 1.37	4.44 ± 1.52	0.659
Hemoglobin (g/L)	112.3 ± 17.2	117.6 ± 15.6	0.282
Elevated transaminase (case)*	12	27	0.035
Serum ceruloplasmin ( μ g/mL)	128.32 ± 13.71	133.02 ± 11.38	0.204
Serum copper ( μ mol/L)	8.33 ± 7.65	11.22 ± 10.01	0.324
24-h urine copper ( μ g)*	527.52 ± 133.29	375.23 ± 93.51	0.000
Ultrasound diagnosis of liver cirrhosis (cases)	11	35	0.509
Ultrasound diagnosis of splenomegaly (cases)	12	38	0.412
Abnormal brain MRI (cases)*	13	44	0.533
The adherence to low-copper diet (cases)*	8	45	0.035
Zinc gluconate dose(mg/d) *	180.22 ± 70.23	321.79 ± 90.87	0.000
Penicillamine dose(mg/d) *	463.19 ± 221.34	625.72 ± 263.28	0.045

^*^The difference between the H-WD group and NH-WD group was statistically significant (*P* < 0.05)

### Laboratory and color doppler examinations

There was no significant difference in the mean hemoglobin, red blood cell count, white blood cell count, and platelet count between the H-WD and the NH-WD groups. The proportion of elevated ALT and AST levels in the NH-WD group was higher than that in the H-WD group (*P* = 0.035). The color Doppler ultrasonography revealed that the number of patients with liver cirrhosis and splenomegaly was not significantly different between the two groups.

### Laboratory data

There was no significant difference in serum copper and serum ceruloplasmin levels between the two groups (*P* = 0.324 and *P* = 0.204, respectively). The adherence to a low-copper diet rate was lower in the NH-WD group than that in the H-WD group (*P* = 0.035). The 24-h urinary copper level in the NH-WD group was higher than that in the H-WD group (*P* = 0.000). Moreover, the average daily oral doses of PCA and ZG in the NH-WD group were lower than those in the H-WD group (*P* = 0.000 and *P* = 0.045, respectively).

### Comparison of MRI of the brain between the H-WD and NH-WD groups

**Fig. 1:**
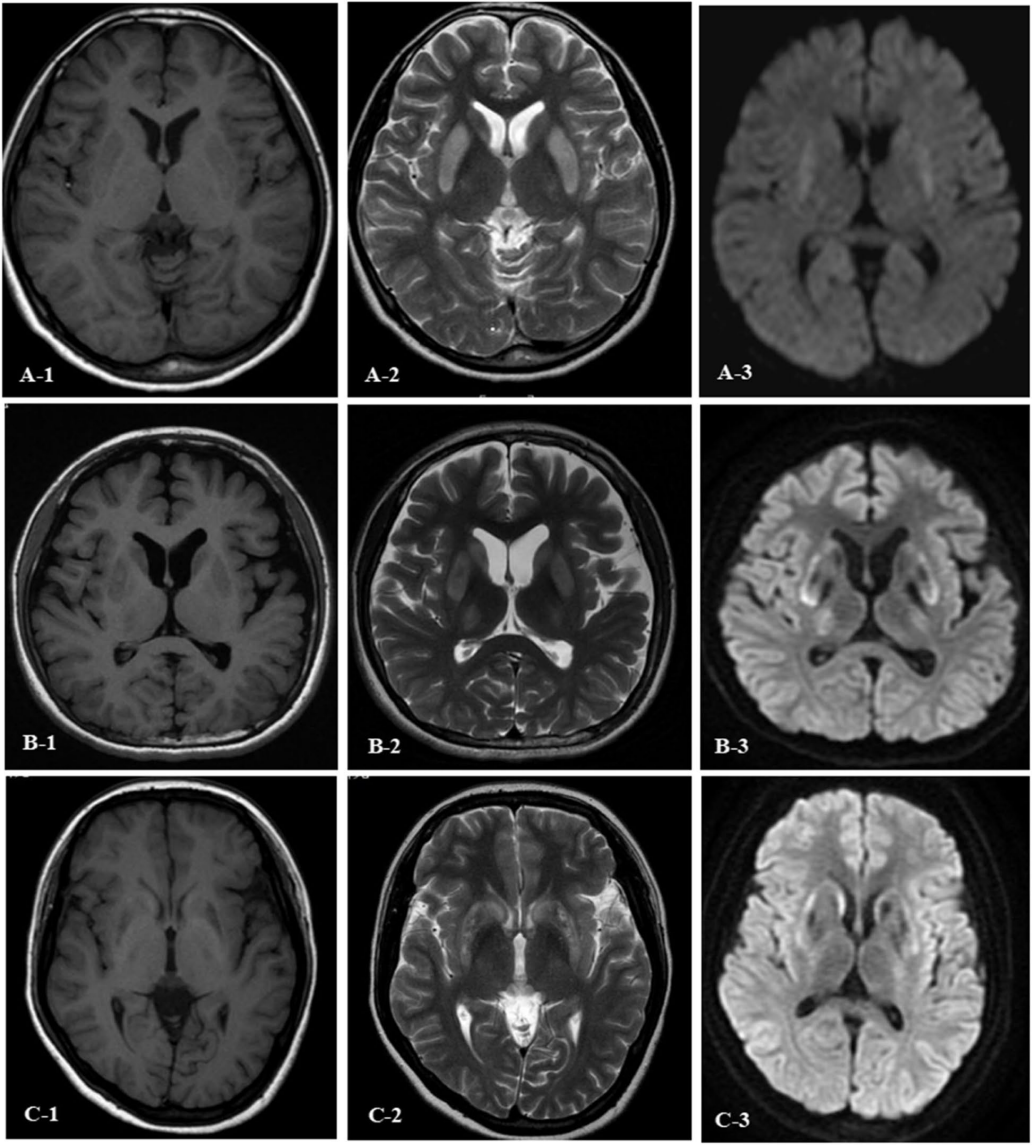
Magnetic resonance imaging (MRI) of the brain in 3 patients with purely hepatic Wilson disease (WD) after appearance of neurological symptoms. A1-A3 images were from a 14-year-old female patient with purely hepatic WD at 3 weeks after appearance of neurological symptoms with the high signal intensity in bilateral putamen and caudate nucleus on T2-weighted imaging (T2WI) and diffusion-weighted imaging (DWI), and the signal intensity was low at the corresponding position on T1-weighted imaging (T1WI). B1-B3 images were from an 18-year-old female patient with purely hepatic WD at 4 weeks after appearance of neurological symptoms, with the high signal intensity (local low signal intensity) in the bilateral putamen, thalamus, and caudate nucleus on T2WI and DWI-MRI, and the signal intensity was low at the corresponding position on T1WI. C1-C3 images were from a 24-year-old female patient with purely hepatic WD at 6 weeks after appearance of neurological symptoms, with the high signal intensity (local mixed signal intensity) in the bilateral putamen and caudate nucleus on T2WI and DWI-MRI, and the low signal intensity at the corresponding position on T1WI

Among WD patients in the NH-WD group, 13 (92.86%) patients had abnormal MRI signals of the brain. MRI of the brain in some NH-WD patients showed a remarkably high signal intensity on T1WI, high signal intensity on T2WI, and high signal intensity on diffusion-weighted imaging (DWI) (Fig. 1). The most common areas of brain damage were globus pallidus, putamen, caudate nucleus, thalamus, midbrain, pons, and cerebellum in 13 (92.86%), 13 (92.86%), 12 (85.71%), 10 (71.43%), 6 (42.86%), 3 (21.43%), and 1 (7.14%) patients, respectively. There were 12 (85.71%) patients with cerebral atrophy.

In the H-WD group, 44 (81.48%) patients had abnormal signals on MRI of the brain, and the common areas of brain damage were globus pallidus, putamen, caudate nucleus, thalamus, midbrain, and pons in 34 (62.96%), 30 (55.56%),18 (33.33%), 15 (27.78%), 6 (11.11%), and 4 (7.41%) patients, respectively. There were 32 (59.26%) patients with cerebral atrophy. There was no significant difference in the abnormal rate of MRI of the brain between the two groups (*P* = 0.533).

### Risk factors associated with neurological symptoms

Univariate analysis showed that elevated ALT and AST levels, 24-h urinary copper level, adherence to a low-copper diet rate, PCA dose, and ZG dose were significantly different between the H-WD and NH-WD groups (Table 1). Considering the occurrence of neurological symptoms as the dependent variable, elevated ALT and AST levels, 24-h urinary copper level, adherence to a low-copper diet rate, ZG dose > 210 mg/day, and PCA dose > 500 mg/day as independent variables, the multivariate logistic regression analysis showed that the low average daily therapeutic doses of PCA and ZG were independent risk factors for the development of neurological symptoms in H-WD patients (Table 2).

**Table 2:** Logistic regression analysis of risk factors for neurological symptoms

Factors	OR Value (ExpB)	95% confidence interval	*P* value
Abnormal transaminase rate	0.252	0.034 ~ 1.858	0.176
24 h urine copper (> 500 μg)	0.765	0.138 ~ 4.232	0.759
The adherence to low-copper diet	3.884	0.679 ~ 22.219	0.127
Zinc gluconate (> 210 mg/day)	8.401	1.739 ~ 40.577	0.008
Penicillamine (> 500 mg/day)	5.42	1.047 ~ 28.07	0.044

## Discussion

It is broadly accepted that WD is an autosomal recessive inherited disorder of copper metabolism. Its clinical and pathological manifestations are the consequences of excessive accumulation of copper in the brain, liver, kidneys, and cornea [8]. The clinical manifestations of WD vary from an asymptomatic state, chronic liver disease, and acute hepatic failure to a neuropsychiatric presentation [9]. Besides, WD is one of the few hereditary diseases that can be successfully treated with medication. Early diagnosis and timely treatment are conducive to effectively controlling WD, whereas undertreatment or poor adherence can be life-threatening [10]. Patients with purely hepatic WD mainly present with unexplained elevated ALT and AST levels, and their age at the time of diagnosis is typically between 5 and 35 years. WD is generally limited to hepatic symptoms without neurological symptoms that can be clinically alleviated with timely treatment [11–13]. In treating patients with purely hepatic WD without neurological symptoms, some patients gradually developed neurological symptoms. Although therapeutic approaches for WD have shown significant advancement, the options for pharmacological treatment options are still limited, especially for WD patients with neurological symptoms [14–16].

What factors cause the development of neurological symptoms in patients with purely hepatic WD? However, there is no unified understanding of this disease. At present, treatment options for patients with WD include medical therapy and liver transplantation. Copper chelators, such as PCA, are the first-line and effective treatment options for WD. Zinc salts, such as ZG, are maintenance drugs for treating WD as they reduce copper absorption from the intestinal tract. PCA and ZG are used in combination as the primary medical treatment for patients with WD [17, 18]. When WD patients with neurological symptoms are treated with PCA, the copper in the body is mobilized to the blood, brain, and other tissues. The copper transported into the brain may lead to cell dysfunction and necrosis, causing the deterioration of neurological symptoms [19]. Studies demonstrated that the deterioration of neurological deterioration occurs during anti-copper treatment, involving the mobilization and redistribution of hepatic copper [19–21]. In recent years, few studies have analyzed the risk factors of early neurological deterioration in neurological WD. These studies were mainly based on clinical and imaging data, such as age, liver cirrhosis, splenomegaly, location of brain MRI lesions in pons and thalami, neurological phenotype, and severe gene mutation types [22–24]. Recent studies have shown that high serum neuro-filament light chain (sNfL) level is a significant risk factor for neurological deterioration, and biochemical detection of sNfL level has been essentially performed for evaluating neurological deterioration in neurological WD [25, 26].

The above-mentioned studies [22–26] mainly aimed to explore the risk factors and assess the deterioration of neurological symptoms in neurological WD. In the present study, all patients with purely hepatic WD were given the combined treatment of PCA and ZG, while 14 (20.59%) of the 68 patients with purely hepatic WD developed neurological symptoms during follow-up. The worsening of neurological symptoms in patients with neurological WD mainly occurs within 2–6 weeks after anti-copper treatment [7]. In the present study, patients with purely hepatic WD did not develop neurological symptoms within a short period (2–6 weeks), and 14 of whom developed neurological symptoms at 0.5–5 years after diagnosis and anti-copper treatment. These patients ruled out the emergence of acute neurological symptoms caused by stress factors, such as traumatic, surgical, or emotional events [27]. It was suggested that there is a significant difference between the risk factors of developed neurological symptoms in purely hepatic WD and the deterioration of neurological symptoms in neurological WD. Neurological symptoms of these patients were either subacute or chronic. It is noteworthy that there may be other factors that cause neurological symptoms in patients with purely hepatic WD.

The results of the MRI of the brain showed that 13(92.86%) patients in the NH-WD group had abnormalities. Among 14 patients in the NH-WD group, it was revealed that 3 patients had low signal intensity on T1WI, and high signal intensity on T2WI and DWI (Fig. 1), indicating that the brain damage in these 3 patients was in the acute or subacute stage [28]. In the H-WD group, no patient with low signal intensity on T1WI and high signal intensity on T2WI and DWI was found, while the proportion of abnormal signals on MRI of the brain was as high as 81.48% (44/54), indicating that copper caused a substantial brain damage and imaging changes in more patients with hepatic WD. Neurological symptoms may occur when brain damage reaches a certain degree. The above-mentioned results indicated that MRI of the brain is of great significance for the assessment of status of patients with H-WD, especially when neurological symptoms occur.

The results of the present study revealed that during the diagnosis and treatment of patients with purely hepatic WD, the elevated ALT and AST levels and 24-h urinary copper level in the NH-WD group were higher than those in the H-WD group, and the adherence to low-copper diet and the average daily oral doses of PCA and ZG in the NH-WD group were lower than those in the H-WD group. Insufficient administration of PCA and ZG in the NH-WD group may prevent copper from being excreted from the body promptly, leading to toxic copper accumulation in the liver, damaging liver cells, and elevated ALT and AST levels. The 24-h urinary copper level in the NH-WD group was also higher, suggesting that the NH-WD group had more copper that was not excreted from the body. This could be related to the insufficient administration of PCA and/or ZG.

Univariate analysis revealed that the adherence to low-copper diet in the NH-WD group was lower than that in the H-WD group. However, logistic regression analysis indicated that the low adherence to low-copper diet was not a high-risk factor in the NH-WD group using the NH-WD group as the control. Recently, several studies have shown that low adherence to low-copper diet is of great significance for controlling damage to target organs caused by copper accumulation [29, 30]. It is still essential to pay further attention to the dietary treatment of patients with purely hepatic WD and reduce the intake of copper to alleviate reduce the damage of copper to target organs, such as the liver and brain.

Logistic regression analysis showed that insufficient doses of PCA and ZG influenced the neurological symptoms of patients with purely hepatic WD. The poor medication adherence may be an important reason for the inadequate dosages of PCA and ZG. In the NH-WD group, a limited number of patients (*n* = 3) could have a strict low-copper diet and a regular oral intake of sufficient doses of PCA and ZG, whereas still developed neurological symptoms. It was found that patients' 24-h urinary copper levels were not high when PCA was used for anti-copper treatment. However, the 24-h urinary copper level was significantly elevated when sodium 2,3-dimercaptopropane-1-sulfonate was used for copper excretion. This suggests that oral PCA treatment for these patients is inadequate for copper excretion. Patients may receive sodium dimercaptosulphonate, dimercaptosuccinic acid, and trientine [31] as copper excretion drugs, and it is recommended to monitor 24-h urinary copper levels to ensure the efficacy of anti-copper treatment. The biomarker sNfL can also be detected to predict the occurrence (or deterioration) of neurological symptoms [25, 26]. Treating patients with purely hepatic WD should follow individualized treatment, and the selection of appropriate and efficient anti-copper drugs is worthy of further investigation [5].

The present study had some limitations. First, the retrospective design and the exclusion of patients with incomplete data might lead to bias. Second, this study was a single-center study with a relatively small sample size, which might hinder the applicability of the finding. Therefore, additional large-scale, multi-center longitudinal studies will be required to eliminate the above-mentioned limitations.

## Conclusions

In conclusion, the development of neurological symptoms in patients with purely hepatic WD was closely associated with the low doses of PCA and ZG, and the poor efficacy of individualized anti-copper therapy. In the anti-copper treatment of patients with purely hepatic WD, it is of great significance to promote patients' educational levels, monitor treatment response, and follow the principle of individualized treatment to prevent the development of neurological symptoms.

## Data Availability

The datasets generated and/or analyzed during the current study are not publicly available because of privacy or ethical restrictions. However, these are available from the corresponding author upon request.
